# Prevalence of Dry Eyes Symptoms in Association with Contact Lenses and Refractive Status in Portugal

**DOI:** 10.3390/life12101656

**Published:** 2022-10-20

**Authors:** Miguel Ángel Sánchez-Tena, Clara Martinez-Perez, Cristina Alvarez-Peregrina, Núcleo de Investigação Aplicada em Ótica e Optometria

**Affiliations:** 1 Department of Optometry and Vision, Faculty of Optics and Optometry, Universidad Complutense de Madrid, 28040 Madrid, Spain; 2 ISEC LISBOA-Instituto Superior de Educação e Ciências, 1750-179 Lisboa, Portugal

**Keywords:** ocular discomfort, CLDEQ-8, dry eye, refractive errors

## Abstract

**Simple Summary:**

The objective of this study is to determine the presence of ocular symptoms in soft-contact-lens wearers that change according to refractive status. To do so, the CLDEQ-8 questionnaire was administered during the months of January to March 2022. Significant differences have been found based on the symptoms present with contact lenses and the degree of myopia. The intensity of visual disturbances was higher in the participants with medium myopia compared to those with low and high myopia. In conclusion, contact-lens users with hyperopia showed a higher rate of ocular dryness than those with myopia. In turn, wearing daily-replacement lenses could be one of the reasons for the lesser presence of ocular dryness when compared to monthly-replacement lenses.

**Abstract:**

Background: Determine whether the presence of ocular symptoms in soft-contact-lens wearers changes depending on the refractive status. Methods: During the months of January to March 2022, the CLDEQ-8 questionnaire was administered to soft-contact-lens wearers. The statistical analysis was carried out using the SPSS 27.0 computer program (SPSS Inc., Chicago, IL, USA). Results: A total of 251 subjects participated in the study, with a higher percentage of myopes than hyperopes (82.1% versus 16.7%; *p* < 0.001). Out of all total participants, 21.5% suffered from dry-eye symptoms. It was noted that hyperopes presented a higher rate of dry-eye symptoms (*p* = 0.041). At the same time, the spherical equivalent was more positive in the participants with dry-eye symptoms (*p* = 0.014). Significant differences were found based on the symptoms present with contact lenses and the degree of myopia. The intensity of visual disturbances was higher in the participants with medium myopia (median [IQR]: 1/5 [2]) compared to those with low (median [IQR]: 0/5 [2]) and high myopia (median [IQR]: 0/5 [1]) (*p* = 0.009). Conclusions: Contact-lens wearers with hyperopia showed a higher rate of ocular dryness than those with myopia. In turn, wearing daily-replacement lenses could be one of the reasons for the lesser presence of ocular dryness compared to monthly-replacement lenses.

## 1. Introduction

Around 140 million people throughout the world wear contact lenses (CL) to correct their refractive errors [1]. Despite the advances that have been made in terms of CL technology, this number has remained stable over the last decade. The main reason for this stability is that 10% to 50% of users stop wearing their CL after 3 years due to discomfort. In fact, 70% of CL wearers report that they experience discomfort by the end of the day, and 40% of soft-CL wearers state that they experience dry eye, 25% of whom report moderate to severe symptoms, resulting in a reduction in the time wearing CL [2,3,4,5,6,7].

In 2013, the Tear Film and Ocular Surface Society (TFOS) defined CL discomfort as “*a condition characterized by episodic or persistent adverse ocular sensations related contact lens wear*”, which is caused by a “*reduced compatibility between the contact lenses and the ocular environment*” [8].

Nowadays, soft CL made from silicone hydrogel are used to correct different types of ametropia. These CL boast a higher oxygen-transmission coefficient than conventional soft CL. The hydrophilic material boasts a good surface-moisturizing capacity, which is sufficient for both tearing and transporting fluids across the CL. Thanks to the specific properties offered, silicone-hydrogel CL offer the best oxygen-transmission rate, therefore making them the most adequate option from a physiological standpoint [9]. However, silicone increases the elasticity of the CL, and some users struggle to tolerate this. In addition, the high oxygen transmissibility activates the peroxidation of proteins in the cornea. Dehydration is one of the main causes of dryness and discomfort. Considering their structure, silicone-hydrogel CL have a high moisture content [9], and the higher water content in the CL results in faster evaporation, with dehydrated conventional soft CL becoming absorbent of the water contained in the tear film. Silicone-hydrogel CL are related to those artificial factors, which lead to a decrease in the tear-film stability in the presence of several factors [10].

Current research on new advances in silicone-hydrogel contact-lens polymers and lens-care products focuses primarily on how to minimize the impact on eye health and increase comfort. The goal is to improve the CL-wearing experience [11,12]. Thus, the new products mainly consider the interaction of the lens with the ocular surface in order to minimize the mechanical and physiological effects on the eye.

Nevertheless, despite all these advances, discomfort caused by dry eye remains the main reason why people choose to discontinue CL wear, and it is the main cause of frustration among both patients and doctors [8].

In 2002, the Contact Lens Dry Eye Questionnaire (CLDEQ) [3,13] was designed and validated to assess dry-eye symptoms among CL wearers. The long version included questions that covered the patient’s CL-wearing history, frequency, use, and presence of ocular-surface symptoms, along with questions about treatments, computer use, and environmental factors.

In the year 2009, an abbreviated version of this questionnaire was developed and validated with eight questions (CLDEQ-8) designed to evaluate the severity of dry-eye symptoms in soft-CL users in the past 2 weeks [14,15]. Each question was answered using a Likert scale of 0 to 4, 0 to 5, or 1 to 6, and the total score was evaluated based on a scale from 0 to 37. The cut-off score for dry-eye diagnosis was established at ≥12 points [15,16].

The objective of this study was to determine whether the presence of ocular symptoms in soft-CL users varied depending on the refractive status.

## 2. Materials and Methods

### 2.1. Study Design and Data Gathering

A prospective, transversal, and observational study was carried out in soft-contact-lens wearers. The CLDEQ-8 questionnaire validated by Chalmers et al. [15] was used as well as questions about demographic, prescription, type, and CL replacement, with the aim of evaluating the severity of dry-eye symptoms in soft-CL wearers in the previous 2 weeks. The cutoff value for dry-eye disease was set to ≥12 points. The supplementary material shows the CLDEQ-8 questionnaire that was used [15].

The data were collected from January to March 2022 throughout the entire region of Lisbon, Portugal.

### 2.2. Statistical Analysis

The statistical analysis was carried out using the SPSS 27.0 computer software (SPSS Inc., Chicago, IL, USA). The normal distribution of the variables was conducted using the Kolmogorov–Smirnov test, establishing a significance level of 0.05. The Wilcoxon rank-sum test was used because of nonparametric distribution. The chi-squared test or Fisher’s exact test were used, as appropriate, to check the association between the categorical variables.

Spearman’s correlation was used for analyzing the quantitative variables. A cutoff point of *p* ≤ 0.05 was considered to assess statistical significance.

To determine the refractive state, the spherical-equivalent formula (SE = SE = sphere + cylinder/2) was used. A patient was considered myopic when the SE was more negative than or equal to −0.50D, hyperopic when it was more positive or equal to +0.50D, and emmetropic when it was between −0.25D and +0.25D [17,18].

The research was conducted in accordance with the principles of the Declaration of Helsinki and with the approval of the ethics committee of the Institute of Education and Science (ISEC Lisbon) on 5 November 2021 recorded under code CE/2022/03/01.

## 3. Results

### 3.1. Demographic Data

A total of 248 subjects participated in the study, with a higher percentage of myopes than hyperopes (83.1% versus 16.9%; *p* < 0.001).

The ages of the participants were 35.22 ± 12.95 years and the median and interquartile range was 34 [20]. With regards to gender, the percentage of women was similar to that of men (56.2% versus 43.8%, respectively; *p* > 0.05). Table 1 shows the demographic data and contact-lens information of the analyzed sample.

### 3.2. CLDEQ-8 Questionnaire

**Figure 1:**
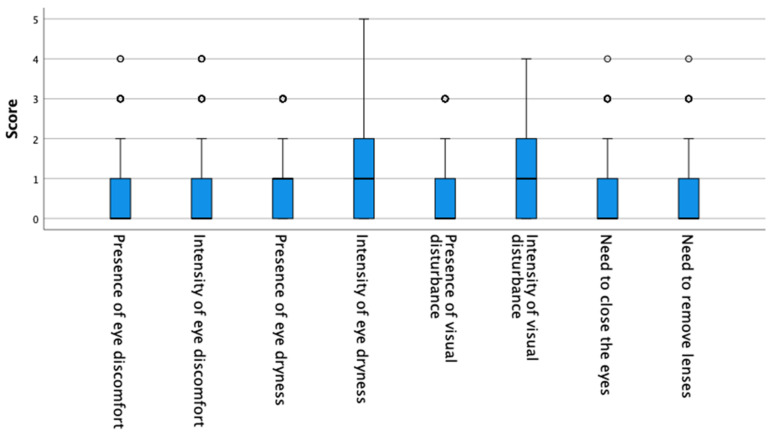
Myopes score on each of the questions of the CLDEQ-8 questionnaire. Box = 1 SD, line = median, whisker = confidence interval 95%, o = extreme values.

Out of all total participants, 21.4% suffered from dry eye. Thus, the median and interquartile range of patients with healthy eyes was 4/37 [7.00] and that of patients with dry eye was 17/37 [7.25]. Among the myopic patients, 81.6% had healthy eyes (median [IQR]: 3.5/37 [7.00]) and 18.4% suffered from dry eye (median [IQR]: 17/37 [6.50]). Figure 1 shows the score of the myopes in each of the questions.

**Figure 2:**
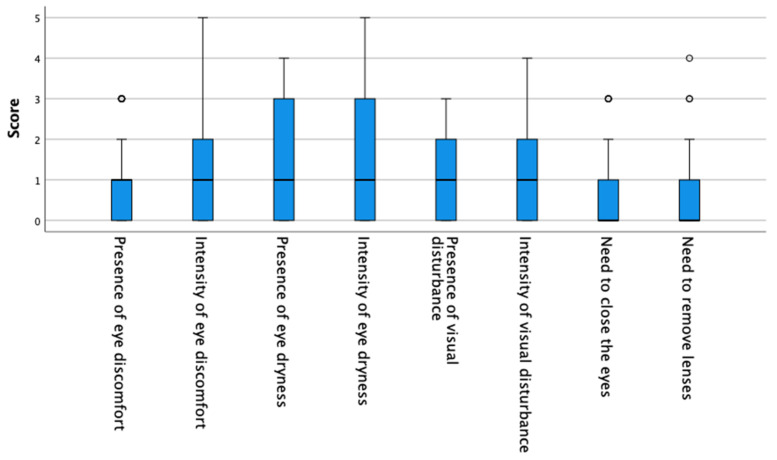
Hyperopes score in each of the questions of CLDEQ-8 questionnaire. Box = 1 SD, line = median, whisker = confidence intervals 95%, o = extreme values.

Among the hyperopic participants, 42.1% had healthy eyes (median [IQR]: 4/37 [6.00]) and 57.9% suffered from dry eye (median [IQR]: 17/37 [9.00]). Figure 2 shows the score of the hyperopes in each of the questions.

When analyzing the frequency of contact-lens replacement, it was found that those who wear daily CL presented less sensation of ocular dryness than wearers of monthly and biweekly CL (*p* = 0.008). In turn, the intensity of ocular discomfort was also lower in daily-replacement CL wearers (*p* = 0.037).

**Figure 3:**
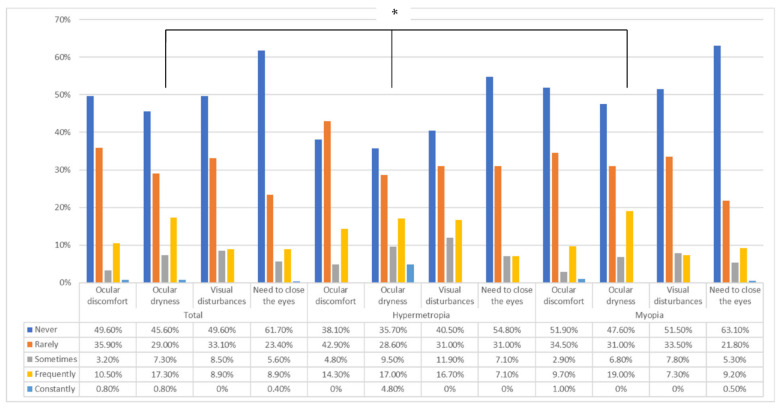
Symptomatology presences in the total number of participants and according to the refractive state. * Significant differences (*p* < 0.05).

When comparing the presence of dry eye based on the refractive state, it was noted that hyperopes presented a higher rate of dry eye (*p* = 0.022). Regarding the presence of other symptoms, no significant differences were found (*p* > 0.05) (Figure 3). Regarding the need to remove the contact lenses, of the total number of participants, 67.7% never needed to, 16.5% less than once a week, 10.1% weekly, 4.8% several times per week, and 0.8% daily. No differences were found based on refractive status, either (*p* > 0.05).

At the same time, the spherical equivalent was more positive in the participants with dry eye (*p* = 0.014). Nevertheless, no significant differences were found in the need to remove CL based on the refractive error (*p* > 0.05).

Figure 3 shows the presence of ocular symptoms depending on the refractive state (emmetropic participants were excluded due to the small sample size).

**Figure 4:**
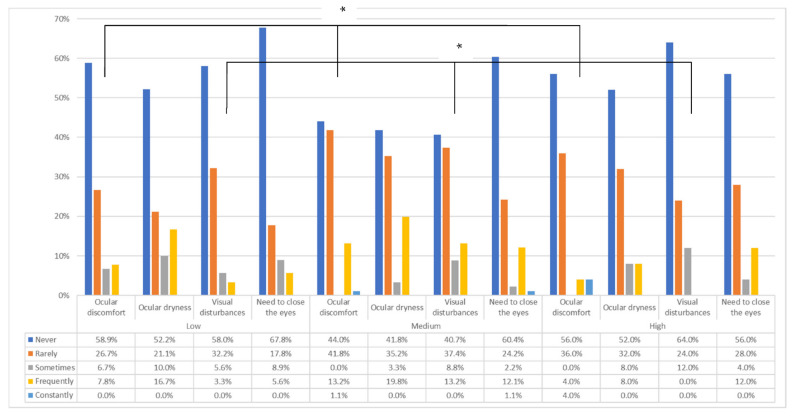
Symptomatology presence according to the degree of myopia. Low: −0.5D < SE > −3D; medium: −3D ≤ SE > −6D; high: SE ≤ −6D. * Significant differences (*p* < 0.05).

Among the participants with myopia, 43.7% (n = 90) had low myopia, 44.2% had medium myopia (n = 91), and 12.1% had high myopia (n = 25). As shown in Figure 4, significant differences were found based on the symptoms present with contact lenses and the degree of myopia. Thus, the presence of symptoms of ocular discomfort (*p* = 0.019) and symptoms of visual disturbances (0.027) were more frequent with moderate myopia. No significant differences were found in the other symptoms (*p* > 0.05). At the same time, the intensity of visual disturbances was higher in the participants with medium myopia (median [IQR]: 1/5 [2]) compared to those with low (median [IQR]: 0/5 [2]) and high myopia (median [IQR]: 0/5 [1]) (*p* = 0.009). Regarding the need to remove contact lenses, there were no significant differences depending on the degree of myopia (*p* > 0.05).

## 4. Discussion

This study showed that the prevalence of dry-eye symptoms in soft-contact-lens wearers in Portugal was 21.4%. This percentage is similar to the results of the study carried out by Vidotti et al. [19], which recorded a prevalence of 27.4% among medical students in Brazil. The results in this study were much higher than those recorded in the study by Abbouda et al. [20] in which a prevalence among Italian teenagers of 9% was recorded, and much lower than those recorded in the studies conducted by García León et al. [21] and Uchino et al. [22], which had a prevalence of 93.9% and 36.1% in university and high school students, respectively. This difference could be explained by the prevalence of refractive errors in the different populations [23,24] or the higher prevalence of high refractive errors in East Asia [25].

Regarding the frequency with which CLs are replaced, in our study we found that monthly and daily CL were the most used. This agrees with the studies by Mohidin et al. [26] and Garcia León et al. [21] in which the most used type of LC was monthly. The high percentage of wearers with daily CL could be due to the great improvements that have been introduced in recent years in terms of materials to ensure better visual quality and improve well-being [27].

In this study, 49.6% of LC wearers declared that they did not experience symptoms of ocular discomfort and 35.9% rarely experienced them. This is in line with the results of the study by Papas et al. [28], in which participants stated that they did not experience symptoms of discomfort within the first 8 h of CL usage. One of the reasons why in our study there were hardly any symptoms of ocular discomfort may be associated with the fact that the levels of discomfort increase when the CL is in contact with the eye for a prolonged period, as the eye seems to “get tired” of wearing the CL, with increased discomfort reported after a prolonged period of use. Given that 40.7% of the participants in our study worn daily CL and that the amount of lens care required was minimal, this could explain why few participants reported symptoms of eye discomfort.

It is worth noting that in our study, as with the symptoms of ocular discomfort, 72% of participants stated that they did not experience, or “rarely” experienced, symptoms of ocular dryness. This, contrasted with the results of the study by Alamri et al. [29], in which 62.2% of participants claimed that they suffered from symptoms of ocular dryness, is in line with the results of the study by Rahmawaty Lubis et al. [30], in which 64.1% of participants stated that they did not experience or “rarely” experienced symptoms of ocular dryness. As previously mentioned, the absence of symptoms of ocular discomfort may be because daily CL replacement could help reduce the symptoms of dry eyes.

At the same time, in our study it was found that 5.6% and 3.3% of the subjects presented occasional and frequent symptoms of visual disturbances, respectively. These results are similar to those of the study by Rahmawaty Lubis et al. [30] and far lower than those recorded in the studies by Mohamad Daud et al. [31] and Sapkota et al. [32]. However, both studies found that these symptoms increased with the use of digital devices; therefore, it could be interesting to consider this point in future studies involving daily-CL wearers to determine the reason for the difference in the symptoms of blurry vision.

On the other hand, 67.8% of the participants in our study did not need to close their eyes or remove the LC. Similar rates were recorded in the study by Mohamad Daud et al. [31]. This could be because our study recorded good compliance in terms of the duration of CL wear. In addition, most participants were aged between 30 and 50 years, which is an age range with a lower rate of corneal infiltrates. It is worth noting that in the study carried out by Rahmawaty Lubis et al. [30], the need to close the eyes or remove the CL was higher. This difference could be explained by the fact that this study was conducted in a location in which it rained almost every day of the month, with fresh and moist conditions.

With regards to the difference between the ocular symptoms based on the refractive state, our study observed a higher prevalence of ocular dryness among hyperopic participants. This went against the results recorded in the study by Alamri et al. [29], in which a higher rate of dryness was recorded amongst myopic patients, and our results agree with the study by Fahmy et al. [33], in which hyperopes presented severe dry eye. Until now, the cause of the presence of dry eye according to the refractive state has not been studied. As for myopia, it is suspected that they may have a drier eye, since the lengthened eye can lead to changes in the ocular surface. However, to compare these results accurately, it would be necessary to know the degree of myopia analyzed in both studies to determine the reason for these discrepancies.

One of the limitations of this study was that the wearing time and the number of years wearing CL were not considered, since the CLDEQ-8 questionnaire does not include those questions. On the other hand, our results are based on a validated questionnaire that measure dry-eye symptomatology, and we did not include any clinical measurement to evaluate dry-eye signs. In future studies, it would be interesting to include these measurements.

## 5. Conclusions

In conclusion, this study found that hyperopic CL wearers experienced a higher level of ocular dryness than myopia participants. In turn, daily-replacement CL wearers had a lower presence of ocular dryness than those with monthly replacement.

## Figures and Tables

**Table 1:** Demographic data and contact-lens information of the study population.

	Total	Hyperopia	Myopia	*p*-Value
**No. of participants**				
(% of the total)	248	42 (16.9%)	206 (83.1%)	*p* < 0.001
**Gender**				0.399
Women	139 (56.0%)	21 (50.0%)	118 (57.3%)	
Men	109 (44.0%)	21 (50.0%)	88 (42.7%)	
**Age**				*p* < 0.001
Mean ± SD	35.25 ± 12.96	45.83 ± 14.02	33.10 ± 11.64	
Median [IQR]	34.00 [21]	48.50 [18]	32.00 [18]	
**SE**				*p* < 0.001
Mean ± SD	−2.65 ± 3.42	2.71 ± 2.43	−3.75 ± 2.42	
Median [IQR]	−2.78 [3.15]	2.12 [1.77]	−3.37 [2.95]	
**CL Replacement**				*p* = 0.045
Daily	101 (40.7%)	19 (45.2%)	82 (39.8%)	
Biweekly	22 (8.7%)	0 (0.0%)	22 (10.7%)	
Monthly	117 (47.2%)	21 (50.0%)	96 (46.6%)	
Quarterly	1 (0.4%)	1 (2.4%)	0 (0.0%)	
Annual	7 (2.8%)	1 (2.4%)	6 (2.9%)	

SD: standard deviation; IRQ: interquartile range; SE: spherical equivalent; CL: contact lens; NA: not available (lack of adequate sample size for statistical analysis).

## Data Availability

Not applicable.
